# Improving healthcare efficiency through long-term care insurance (LTCI): a super-SBM and DID analysis of 291 Chinese cities

**DOI:** 10.3389/fpubh.2025.1619791

**Published:** 2025-07-21

**Authors:** Tongtong Jin, Ayitijiang Halili

**Affiliations:** ^1^School of Law, Shanxi University of Finance and Economics, Taiyuan, China; ^2^College of Public Management (Law), Xinjiang Agricultural University, Urumqi, China

**Keywords:** long-term care insurance, healthcare efficiency, super-SBM model, difference-indifferences, population aging

## Abstract

**Background:**

The intensification of population aging has exacerbated the strain on medical resources. Long-term care insurance (LTCI) influences healthcare efficiency by redefining the boundaries between medical and care services. However, its mechanisms and effectiveness in developing countries remain underexplored. This study investigates the impact pathways and heterogeneous characteristics of the effects of LTCI on regional healthcare efficiency in China, providing evidence for policy optimization.

**Methods:**

Using panel data from 291 prefecture-level cities in China from 2010–2021, healthcare efficiency was measured via the slack-based measure super efficiency (Super-SBM) model. The difference-in-differences (DID) method was employed to evaluate the policy effects of LTCI. Bootstrap-based mediation models were used to examine the transmission mechanisms of hospitalization volume, average length of stay, and the number of care institutions. Regional heterogeneity was also analyzed.

**Results:**

LTCI significantly improved regional healthcare efficiency (β = 0.071, *P* < 0.01). Mechanism analysis identified three effective pathways: (1) Reducing hospitalization demand (λ = −0.419, *P* < 0.01) freed up medical resources, contributing 3.42% of the efficiency gains; (2) Shortening length of hospital stay (λ = −0.326, *P* < 0.01) accelerated bed turnover, accounting for 47.6% of the total effect, making it the dominant pathway; (3) Expanding institutional care supply (λ = 0.330, *P* < 0.05) diverted patient flows, explaining 9.23% of the improvement. Heterogeneity analysis indicated that the policy effects were more pronounced in the eastern and central regions and new first- and third-tier cities.

**Conclusions:**

LTCI is an effective tool for optimizing the allocation of medical resources. Region-specific strategies should be adopted to increase demand-side incentives and advance supply-side reforms. This study provides new insights for the efficient utilization of medical resources and the design of LTCI systems in developing countries.

## 1 Introduction

Regional healthcare efficiency serves as a pivotal metric for evaluating the performance and sustainability of healthcare systems (1, 2). Globally, health systems face persistent challenges in hospital bed resource misallocation, characterized by the prolonged occupancy of acute care beds by patients requiring long-term care. In Ontario, Canada, 16% of acute care beds are occupied by alternative levels of care patients, 63% of whom require long-term care placement (3). This phenomenon manifests as a dual dilemma in China, where accelerated population aging exacerbates systemic inefficiencies. On the demand side, 38.1% of disabled older adults aged over 60 years' experience limitations in activities of daily living, whereas 23.2% require continuous professional care (4). On the supply side, tertiary hospitals maintain bed occupancy rates exceeding 90%, in contrast with the 40% vacancy rates reported in primary care facilities (5). This structural imbalance has precipitated a medicalization trap. When long-term care services are covered by health insurance, patients increasingly opt for costlier inpatient care over specialized long-term care services, perpetuating resource misallocation.

International evidence has demonstrated that establishing long-term care insurance (LTCI) systems effectively addresses these systemic challenges. Following LTCI implementation in the Netherlands (1968), Germany (1995), and Japan (2000) (6–8), acute hospital care has been progressively substituted by more cost-effective care institutions, leading to measurable reductions in hospitalization rates (9). Although these systems exhibit heterogeneous financing models (as shown in Table 1), they share three defining characteristics: (1) clear delineation of coverage boundaries between medical and long-term care services; (2) establishment of independent financing mechanisms to prevent medical fund crowding-out; and (3) utilization of payment reforms to incentivize service diversion to primary care facilities. China's LTCI pilot program, initiated in 2016, has been progressively implemented across 49 cities, with a projected coverage of 180 million enrollees by 2025 (10).

**Table 1:** LTCI in different countries.

**Area**	**Financing**	**Service items**	**Service form**
Netherlands	Compulsory premium, copayment, general	Social support, health care, and specialized medical treatments	Home care, day care, community care, institutional care
Germany	Employer-employee contributions, national subsidies	Basic daily living assistance, rehabilitation care	Home care, residential care, semi-residential care, short-term care
Japan	Insurance premiums, government subsidies, and user fees	Basic daily living assistance, social support, rehabilitation care	Home care, institutional care

The table content is organized according to Dai (73).

The literature has extensively explored the policy effects of LTCI across multiple dimensions. From an economic perspective, the prevailing view suggests that LTCI reduces healthcare expenditures by substituting high-cost medical services. Chung and Shil (2015) reported that nonusers of LTCI experienced 87.31 additional hospital days annually, with associated medical costs increasing by 5,449,000 KRW per year compared with those of users (11). Chinese scholars have further validated its crowding-out effect on medical insurance expenditures (12, 13), although significant urban–rural heterogeneity exists, with cost-containment effects primarily observed in urban areas (14). However, some studies challenge this view. Liu and Hu (15) argued that LTCI might increase total healthcare expenditures by expanding service coverage. For health outcomes, a consensus has emerged on the positive impact of LTCI on the well being of disabled older adults (16–18). Some scholars, focusing on caregiver perspectives, highlight that LTCI implementation alleviates physical and mental health burdens among service providers, with sustained long-term health improvements (19, 20). Despite these advancements, three limitations persist in the literature: (1) Fragmented research perspectives. Microlevel individual studies fail to capture the systemic impact of the LTCI on regional healthcare resource allocation. (2) Methodological constraints. Traditional Data Envelopment Analysis (DEA) models inadequately handle undesirable outputs and slack variables, leading to systematic biases in healthcare efficiency measurement. (3) Partial mechanism explanations. Existing studies predominantly focus on demand-side substitution effects while neglecting the supply-side structural incentives induced by insurance payment mechanisms.

This study constructs a dual-pathway analytical framework that integrates demand substitution and supply induction. By utilizing panel data from Chinese prefecture-level cities (2010–2021), we employ the slack-based measure super efficiency (Super-SBM) model, difference-in-differences (DID) method, and mediation effect models to address three research questions: (1) Does the LTCI improve regional healthcare efficiency? (2) Through what mechanisms does LTCI enhance healthcare efficiency by coordinating demand-side and supply-side factors? (3) Does policy effectiveness exhibit regional heterogeneity? This study advances the literature in three ways. First, it empirically demonstrates the efficiency-enhancing effects of LTCI within a developing country context, complementing existing European findings. Second, we advance the field by applying the Super-SBM model to address traditional DEA's efficiency overestimation bias. Third, we reveal the dual-pathway mechanism through which LTCI improves healthcare efficiency, via demand substitution and supply inducement-thereby providing a theoretical foundation for achieving comprehensive LTCI coverage.

## 2 Theory and hypotheses

### 2.1 Dual-pathway mechanism of the impact of LTCI on regional healthcare efficiency

**Figure 1:**
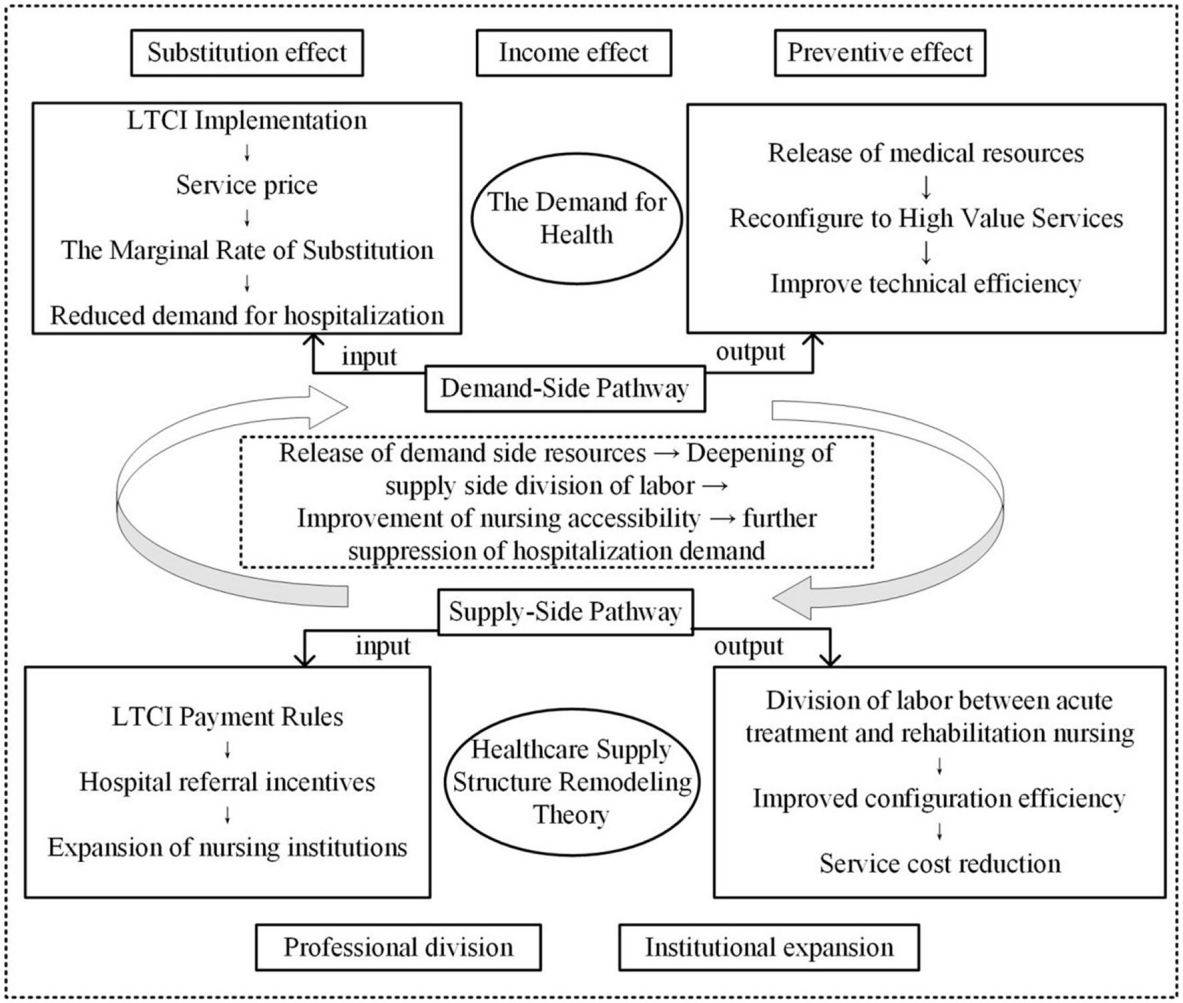
Dynamic synergy between the demand-side pathway and supply-side pathway.

This study constructs a dual-pathway model integrating demand substitution and supply inducement analytical framework (as shown in Figure 1), grounded in Grossman's (21) health demand theory and Arrow's (22) healthcare supply structure remodeling theory. This framework systematically elucidates the mechanisms through which LTCI influences regional healthcare efficiency. Demand-side pathway: Grossman's (21) health demand model posits that individuals optimize their health capital stock by selecting combinations of medical and care services. The LTCI reduces inefficient hospitalization demand through three mechanisms: (1) Substitution effect. Community-based care services covered by LTCI directly replace unnecessary long-term hospitalizations (23). LTCI reduces the relative price of nursing services, thereby increasing their marginal rate of substitution. This incentivizes patients to opt for more cost-effective care alternatives. (2) Income effect. LTCI reimbursement alleviates out-of-pocket financial burdens (24), raising disposable income and relaxing household budget constraints. Consequently, it reduces excessive hospitalization behaviors induced by catastrophic medical expenditures. (3) Preventive effect. LTCI health management services (e.g., regular assessments, chronic disease monitoring) reduce complication risks, decreasing acute medical demand (25).

Supply-side pathway: Arrow's (22) healthcare supply structure remodeling theory highlights that healthcare markets are uncertain and that payment rules can shape provider behavior. As a third-party payer, LTCI induces supply expansion through risk-sharing mechanisms, ultimately reshaping healthcare resource allocation. The LTCI optimizes resource allocation via two mechanisms: (1) Functional specialization. Hospitals focus on acute care (shortening hospital stays), whereas care institutions assume rehabilitation services (improving bed turnover rates) (26, 27). The payment mechanisms of LTCI (e.g., per-diem reimbursement) impose binding constraints on post-acute referrals, incentivizing hospitals to reduce length of stay. Concurrently, care institutions assume responsibility for rehabilitative care, establishing a well-coordinated division of labor between acute treatment and post-acute rehabilitation. (2) Institutional expansion. LTCI's guaranteed payment structure (e.g., fee-for-service models) mitigates operational risks for care institutions, attracting private capital investment. This expansion fosters economies of scale, thereby reducing per-unit service costs (28).

The reduction in hospitalizations on the demand side liberates bed resources, which dynamically synergizes with supply-side specialization to enhance healthcare efficiency through three distinct mechanisms. (1) Resource reallocation effect. Hospitals redirect surplus bed capacity to higher-value services (e.g., intensive care), thereby improving technical efficiency. (2) Learning curve effect. Care institutions develop rehabilitation expertise through service accumulation, leading to enhanced allocative efficiency. (3) Network externality effect. Increased density of care facilities improves service accessibility, subsequently strengthening the elasticity of demand substitution for post-acute care services.

### 2.2 Hypothesis development

Grossman's health production function conceptualizes healthcare demand as an investment in health capital. The LTCI restructures demand in three dimensions: (1) Price effect. Reducing the cost of care services increases their marginal substitution rate relative to hospitalization services (29). (2) Budget effect. Insurance reimbursement alleviates household budget constraints, enabling greater allocation of resources to preventive care within health investment portfolios. (3) Depreciation effect. Health management services extend the depreciation cycle of health capital, reducing the frequency of acute medical demands (30). Concurrently, Arrow's healthcare supply structure remodeling theory reveals that LTCI payment rules reshape provider incentives, inducing a supply-side structural transformation characterized by specialization in acute care and economies of scale in rehabilitation services (31, 32). This synergistic evolution of demand- and supply-side mechanisms drives systemic improvements in regional healthcare efficiency. The specific correspondence between theoretical constructs and research hypotheses is presented in Table 2.

*H1*: LTCI significantly enhances regional healthcare efficiency.

**Table 2:** Theoretical and hypothesis correspondence.

**Hypotheses**	**Theoretical mechanism**
H1	Dual path collaboration enhances technical efficiency and configuration efficiency
H2	Substitution effect and income effect reduce hospitalization demand
H3	Payment rule constraints shorten the hospitalization period
H4	Risk-sharing incentives for care institution expansion

LTCI reduces hospitalization rates through both price and income effects. First, the price effect operates through LTCI's reimbursement of nursing service costs, directly lowering their relative price. According to the intertemporal elasticity of substitution in the Grossman model (21), this price reduction significantly increases the substitution of nursing services for hospitalization. Second, the income effect mitigates hospitalization behaviors induced by catastrophic health expenditures by reducing out-of-pocket payment ratios (33). The bed resources freed by reduced hospitalization rates are reallocated to higher-value medical services, creating a Pareto structural adjustment that significantly enhances regional healthcare efficiency by reducing congestion costs within the healthcare system. Importantly, this observed reduction in hospitalization rates is not attributable to concurrent healthcare reforms such as tiered healthcare policies. While tiered policies may redirect patient flows to primary care facilities by adjusting service levels, their mechanism fundamentally differs from LTCI's demand-side restructuring through price signals. Tiered policies primarily affect service accessibility rather than alleviating household financial risks, representing distinct theoretical foundations and policy objectives (34).

*H2*: LTCI improves regional healthcare efficiency by reducing hospitalization rates.

The contractual binding force of LTCI payment rules plays a pivotal role in healthcare utilization patterns. Specifically, reimbursement terms (e.g., per-diem payment) establish exogenously imposed institutional constraints by contractually defining the maximum duration for acute-phase treatment. Healthcare institutions exceeding these limits face financial disincentives through reduced reimbursement rates or outright payment denials. Concurrently, the path dependency of learning effects manifests as LTCI-induced specialization in acute care generates cumulative learning curves. Hospitals progressively enhance their technical proficiency in specific therapeutic domains over time, leading to organic reductions in treatment duration (35). Combining the exogenous constraints of LTCI payment regulations with endogenous specialization effects, this dual mechanism ultimately produces the observed compression of hospitalization periods. The institutional framework creates immediate financial boundaries, while the learning process ensures sustained efficiency improvements, demonstrating how policy design can simultaneously impose constraints and incentivize performance optimization within healthcare delivery systems.

*H3*: LTCI improves regional healthcare efficiency by reducing the average length of hospital stay.

The LTCI's payment commitment reduces market entry risk for care institutions through two mechanisms. First, the stability of payment commitments, particularly through fee-for-service reimbursement models, provides long-term cash flow security that substantially reduces sunk cost risks for private capital investments (36). Second, a positive feedback loop emerges between service density and demand elasticity: increased spatial density of care facilities enhances service accessibility, which in turn amplifies the substitution elasticity of demand. This creates a self-reinforcing cycle where supply expansion facilitates demand migration, which subsequently stimulates further supply growth. The institutional guarantee of stable payments lowers entry barriers, while the network effects of geographic concentration generate endogenous growth momentum, collectively explaining the observed market expansion dynamics under LTCI implementation.

*H4*: LTCI improves regional healthcare efficiency through the increased availability of care institutions.

## 3 Data, variables, and method

### 3.1 Data sources

This study utilizes data from the China Statistical Yearbook and the EPS database. For cities with missing data on healthcare resources and medical service utilization, we manually collected supplementary statistics from regional health commission reports to ensure completeness. For the remaining small portion of unavailable data, interpolation methods were applied to maintain data integrity and continuity. The study period spans from 2010 to 2021. Following the 2016 implementation of the LTCI pilot policy, the policy-affected regions constitute the treatment group, and the other regions constitute the control group. Data processing was conducted via Excel and Stata 18.0, which involved rigorous cleaning, organization, and analysis of the raw data. Through this meticulous process, we obtained a final dataset comprising 3,349 observations across 291 prefecture-level cities in 31 provinces. The dataset includes 389 treatment group observations and 2,960 control group observations, providing a robust foundation for subsequent empirical analysis.

### 3.2 Variables

#### 3.2.1 Dependent variable

Regional healthcare efficiency is measured via the variable returns to scale Super-SBM model, which addresses heterogeneous resource distributions and scale effect variations in developing countries' medical systems (37) (Equation 1). For the selection of input indicators in healthcare efficiency measurement, we draw on existing research (38, 39) and categorize inputs into three dimensions: human resources, material resources, and financial resources. Output indicators are selected based on both health outcomes and economic benefits. The specific indicators are detailed in Table 3. To ensure robustness, we measure regional healthcare efficiency via the Super-SBM model under constant returns to scale as an alternative approach. To further account for the dimension of healthcare service quality, this study incorporates insights from research on medical resource overutilization (40). We substitute the undesirable output indicator with the annual hospitalization rate and subsequently recalculate healthcare service efficiency using the Super-SBM model.

**Table 3:** Indicator system for regional healthcare efficiency.

**Indicator type**	**Indicator name**	**Indicator content**
Inputs	Number of hospitals	Number of hospitals and health centers processed logarithmically
	Number of hospital beds	The number of beds in hospitals and clinics
	Number of doctors	Total number of doctors
	Medical and health expenditures	Financial medical expenses/financial expenditures
Desirable outputs	Number of discharged patients	Total number of discharged patients
	Frequency of diagnosis and treatment	Outpatient reception capacity
Undesirable outputs	Per capita medical expenditure	Per capita healthcare expenditure of households

#### 3.2.2 The explanatory variable

Drawing on previous research (41), we employ a dummy variable (treat) indicating whether LTCI has been implemented as the explanatory variable. We select enrollees of basic employee medical insurance from 20 cities, including Binzhou, Liaocheng, and Jinan; enrollees of both employees and resident medical insurance from Tonghua, Songyuan, Baishan, Changchun, and Jilin; and enrollees of employees and urban–rural resident medical insurance from nine cities, including Qingdao, Rizhao, and Dongying, as the treatment group. The control group consisted of populations not covered by the LTCI. To evaluate the policy's impact, we introduce a time variable (after), assigning a value of 1 to years following the implementation of the LTCI pilot policy (2016 and later) and 0 to years before implementation.

#### 3.2.3 Control variables

Given that the selection of LTCI pilot cities was not randomized, we draw on literature (42, 43) to select control variables from three dimensions: economic, policy, and demographic characteristics. Economic characteristics include: (1) Economic development level measured by the natural logarithm of per capita GDP. (2) Industrial structure is measured as the ratio of the non-primary sector to GDP. (3) Urbanization level measured as the proportion of urban residents to the total population. Policy-related characteristics include: (1) Government fiscal expenditure, measured as the ratio of fiscal expenditure to GDP. (2) Openness to trade is measured as the ratio of total import and export value to GDP. The demographic characteristics include the following: (1) Population density measured by the natural logarithm of the population per square kilometer. (2) Education level is measured as the ratio of total higher education enrollment to the regional population. For robustness checks, we consider the hierarchical medical system policy, which is coded as 1 for pilot implementation cities and 0 otherwise. To address potential omitted variable bias, we incorporate general public budget health expenditure as an additional control variable in robustness tests. This indicator quantifies the intensity of local governmental fiscal investment in the healthcare system.

#### 3.2.4 Mediating variables

Consistent with the theoretical framework, the mediating variables are selected from the demand and supply sides. The demand-side variable is the number of hospitalizations, measured by the natural logarithm of total hospital admissions. The supply side includes two variables: the average length of hospital stays, measured by the mean duration of hospitalization, and the number of care institutions, measured by the natural logarithms of the number of beds in older adult care institutions.

### 3.3 Methods

#### 3.3.1 Super-SBM model

To address the limitations of traditional DEA models, this study employs the Super-SBM model. This model incorporates slack variables, enabling more nuanced differentiation among inefficient decision-making units (DMUs). Here, 291 Chinese prefecture-level cities (2009–2021), where each city-year pair (i,t) is treated as a unique DMU to capture time-varying efficiency. This setup allows us to capture the time-varying characteristics of healthcare efficiency, as each DMU reflects the operational state of a city in a specific year. For each DMU (i,t), the Super-SBM model calculates efficiency by integrating three components: (1) Input variables ${\;x}_{li}^{t}$: represent healthcare resources, such as the number of hospitals, hospital beds, doctors, and medical and health expenditures of city i in year t. (2) Desirable outputs $y_{ri}^{t}$: reflect health service achievements, including the number of discharged patients and frequency of diagnosis and treatment in city i during year t. (3) Undesirable outputs $b_{ki}^{t}$: indicate negative outcomes, e.g., per capita medical expenditures of city i in year t (detailed in Table 3). The model uses slack variables $s_{i}^{-}$, $s_{r}^{+}$, $s_{k}^{-}$to measure inefficiencies in inputs and outputs, then applies an optimization algorithm to minimize the efficiency loss ratio. Through this process, it generates the efficiency score $\rho_{it}^{*}$, which quantifies the relative efficiency of city i in year t, reflecting both regional (across cities i) and temporal (across years t) variations. The equation is constructed as follows (44, 45):


(1) $$\begin{array}{l} \;\rho_{it}^{*}=\min_{\text{λ},s^{-},s^{+}}\frac{1+\frac{1}{m}\sum_{l=1}^{m}\frac{s_{l}^{-}}{x_{li}^{t}}}{1-\frac{1}{q+h}\left(\sum_{r=1}^{q}\frac{s_{r}^{+}}{y_{ri}^{t}}+\sum_{k=1}^{h}\frac{s_{k}^{-}}{b_{ki}^{t}}\right)}\\ s.t.\;{\;x}_{li}^{t}\geq \sum_{t=1}^{T}\sum_{j=1,j\neq i}^{n}{\text{λ}}_{j}^{t}x_{lj}^{t}-s_{l}^{-}\;\\ \;l=1,2,\ldots ,m;\;t=1,2,\ldots ,T;\\ \;y_{ri}^{t}\leq \sum_{t=1}^{T}\sum_{j=1,j\neq i}^{n}{\text{λ}}_{j}^{t}y_{rj}^{t}+s_{r}^{+}\;\\ \;r=1,2,\ldots ,q;\;t=1,2,\ldots ,T;\\ \;b_{ki}^{t}\geq \sum_{t=1}^{T}\sum_{j=1,j\neq i}^{n}{\text{λ}}_{j}^{t}b_{kj}^{t}-s_{k}^{-}\;\\ \;k=1,2,\ldots ,h;\;t=1,2,\ldots ,T;\\ {\text{λ}}_{j}^{t}\geq 0\left(\forall j\right),s_{l}^{-}\geq 0\left(\forall l\right),s_{r}^{+}\geq 0\left(\forall r\right),s_{k}^{-}\geq 0\left(\forall k\right). \end{array}$$

In the model, $\rho_{it}^{*}$ denotes the efficiency score of DMU i (the i-th city) in year t, ranging between 0 and 1. T = 12 years (2010–2021), with t explicitly indexed to match the panel data structure. *l* denotes the number of input variables, with values ranging from *l* to m; r represents the number of desirable output variables, taking values from 1 to q; k indicates the number of undesirable output variables, ranging from 1 to h; $s_{l}^{-}$ corresponds to the slack variable of inputs; $s_{r}^{+}$ refers to the slack variable of desirable outputs; $s_{k}^{-}$ designates the slack variable of undesirable outputs; λ represents the weight variable. Inputs ($x_{li}^{t}$) are healthcare resources (e.g., number of hospitals, hospital beds, doctors, and medical and health expenditures) for city i in year t. Desirable outputs ($y_{ri}^{t}$) are health service outputs (e.g., number of discharged patients and frequency of diagnosis and treatment). Undesirable outputs ($b_{ki}^{t}$) are per capita medical expenditures (as shown in Table 3). The model evaluates healthcare efficiency dynamics under China's LTCI policy, where DMUs are stratified into treatment (pilot cities post-2016) and control groups. The efficiency scores $\rho_{it}^{*}$ serve as the dependent variable in subsequent DID analysis, reflecting annual variations in regional efficiency.

#### 3.3.2 DID model

To effectively identify the impact of the LTCI on regional healthcare efficiency, this study treats the LTCI pilot policy as a quasi-natural experiment, assessing its policy effects through causal inference. Drawing on previous research (46), we construct a DID model:


(2) $$\begin{array}{l} MEFF_{i,t}=\beta_{0}+\beta_{1}LTCI_{i,t}+\beta_{2}X_{i,t}+\gamma_{i}+\delta_{t}+\varepsilon_{i,t} \end{array}$$

In the model, i denotes the region index, specifically representing prefecture-level city regions in China (corresponding to the 291 prefecture-level city samples mentioned earlier), and t represents time. *MEFF*_*i, t*_ is the dependent variable, representing regional healthcare efficiency. *LTCI*_*i, t*_ is the core explanatory variable, indicating whether the LTCI has been implemented in the region. *X*_*i, t*_ represents a set of control variables, γ_*i*_ captures region fixed effects, δ_*t*_ captures time fixed effects, and ε_*i, t*_ is the random error term.

#### 3.3.3 Mediating effect model

To identify the mechanisms underlying the impact of LTCI on regional healthcare efficiency, we adopt a two-stage mediating effect model. We first estimate the effect of LTCI on mediating variables, then examine how these mediators transmit influences to healthcare efficiency. The empirical specifications are as follows:


(3) $$\begin{array}{l} M_{i,t}^{*}={\text{λ}}_{0}+{\text{λ}}_{1}LTCI_{i,t}+{\text{λ}}_{2}X_{i,t}+\gamma_{i}+\delta_{t}+\varepsilon_{i,t} \end{array}$$

(4) $$\begin{array}{l} MEFF_{i,t}=\eta_{0}+\eta_{1}LTCI_{i,t}+\eta_{2}M_{i,t}^{*}+\eta_{3}X_{i,t}+\gamma_{i}+\delta_{t}+\varepsilon_{i,t} \end{array}$$

The mediating variables ($M_{i,t}^{*}$) include the number of hospitalizations (hosp), average length of hospital stay (hospdate), and number of care institutions (careco). Key coefficients have specific meanings: λ_1_ is the effect of LTCI on mediating variables, η_1_ is the direct effect of LTCI on efficiency, η_2_ is the effect of mediators on efficiency, the indirect effect is λ_1_ × η_2_, and the total effect is their sum. For mediating effect testing, if λ_1_ and η_2_ are significant, partial mediation exists if η_1_ is significant, and complete mediation exists if η_1_ is not; if λ_1_ or η_2_ is non-significant, we use the Bootstrap method to test λ_1_ #x000D7; η_2_. The definitions of the other parameters remain consistent with Equation 2.

#### 3.3.4 Parallel trends test model

To validate the causal effects of policy implementation, we employ an event study approach to examine the dynamic trends in regional healthcare efficiency before and after the introduction of the LTCI pilot policy. Drawing on previous research (47), we construct the model:


(5) $$\begin{array}{l} MEFF_{i,t}=\alpha +\overset{5}{\underset{\tau =-6}{\sum }}\beta_{\tau }EVENT_{\tau ,i,t}+\text{λ}{\text{X}}_{i,t}+{\text{γ}}_{i}+{\text{δ}}_{t}+{\text{ε}}_{i,t} \end{array}$$

*EVENT*_τ, *i, t*_ is the event study dummy variable. τ = t – t0 (where t0 denotes the implementation year of the LTCI policy; τ < 0 represents the pre-policy period, and τ ≥ 0 represents the post-policy period). The base period can be set as τ = −1, i.e., 1 year before policy implementation. β_τ_ measures the difference in medical efficiency between the treatment and control groups relative to the base period in the τ period before/after policy implementation. If the statistics for the pre-policy period (τ < 0) are not significant and fluctuate around zero, it indicates that the parallel trends assumption is satisfied.

## 4 Results

### 4.1 Descriptive statistics

Table 4 presents the descriptive statistics of the main variables. The mean value of the dependent variable, regional healthcare efficiency, is 0.403 (SD = 0.189), indicating substantial regional variation in healthcare efficiency and an overall relatively low efficiency level. The mean value of the explanatory variable, the LTCI, is 0.047 (SD = 0.212), suggesting low coverage of the LTCI and significant regional disparities.

**Table 4:** Descriptive statistics.

**Variable**	**Mean**	**SD**	**Min**	**Max**
Healthcare efficiency	0.403	0.189	0.056	1
LTCI	0.047	0.212	0	1
Hospitalizations	13.176	0.766	10.461	15.641
Hospital stays	8.839	1.538	3.225	13.893
Care institutions	9.387	1.094	2.966	14.959
Economic development level	10.699	0.589	8.576	13.056
Population density	5.763	0.909	1.609	7.882
Industrial structure	0.878	0.078	0.501	1
Government fiscal expenditure	0.202	0.11	0.044	1.485
Education level	0.02	0.026	0	0.223
Openness to trade	0.203	0.345	0	3.858
Urbanization level	0.552	0.152	0.171	1
Hierarchical medical system policy	0.414	0.493	0	1
General public budget health expenditure	2.741	1.361	0.104	6.452

### 4.2 Main regression results

Table 5 presents regression results across progressively controlled specifications. Column (1) employs region-fixed effects, Column (2) adds time-fixed effects, Column (3) introduces control variables with region-fixed effects, and Column (4) combines two-way fixed effects with full controls. The column (5) represents the 95% confidence interval of the regression results from the column (4). LTCI significantly improves regional healthcare efficiency (β = 0.071, *P* < 0.01) across all specifications, providing robust support for H1.

**Table 5:** Difference-in-differences (DID) estimation results.

**Variable**	**(1) (95% CI)**	**(2) (95% CI)**	**(3) (95% CI)**	**(4) (95% CI)**
LTCI	0.074^***^ (0.041 to 0.106)	0.062^***^ (0.027 to 0.096)	0.094^***^ (0.061 to 0.126)	0.071^***^ (0.038 to 0.105)
	(0.017)	(0.018)	(0.016)	(0.017)
Economic development level			−0.028^*^ (−0.058 to 0.002)	0.062^**^ (0.005 to 0.119)
			(0.015)	(0.029)
Population density			−0.079 (−0.266 to 0.108)	−0.062 (−0.248 to 0.124)
			(0.095)	(0.095)
Industrial structure			0.992^***^ (0.697 to 1.287)	0.394^**^ (0.072 to 0.716)
			(0.150)	(0.164)
Government fiscal expenditure			0.173^**^ (0.006 to 0.34)	0.268^*^ (−0.013 to 0.55)
			(0.085)	(0.143)
Education level			−0.010 (−1.093 to 1.072)	0.131 (−0.952 to 1.215)
			(0.550)	(0.551)
Openness to trade			0.108^***^ (0.047 to 0.17)	0.087^***^ (0.024 to 0.149)
			(0.031)	(0.032)
Urbanization level			−0.139^**^ (−0.251 to −0.026)	0.0300 (−0.101 to 0.161)
			(0.057)	(0.067)
Constant	0.400^***^ (0.398 to 0.401)	0.444^***^ (0.429 to 0.459)	0.302 (−0.833 to 1.438)	−0.264 (−1.571 to 1.043)
	(0.001)	(0.008)	(0.577)	(0.664)
City	Yes	Yes	Yes	Yes
Year	No	Yes	No	Yes
*N*	3,349	3,349	3,349	3,349
*R^2^*	0.015	0.137	0.062	0.166

Standard errors in parentheses; ^*^p < 0.1, ^**^p < 0.05, ^***^p < 0.01. The following table is the same.

### 4.3 Parallel trends test

**Figure 2:**
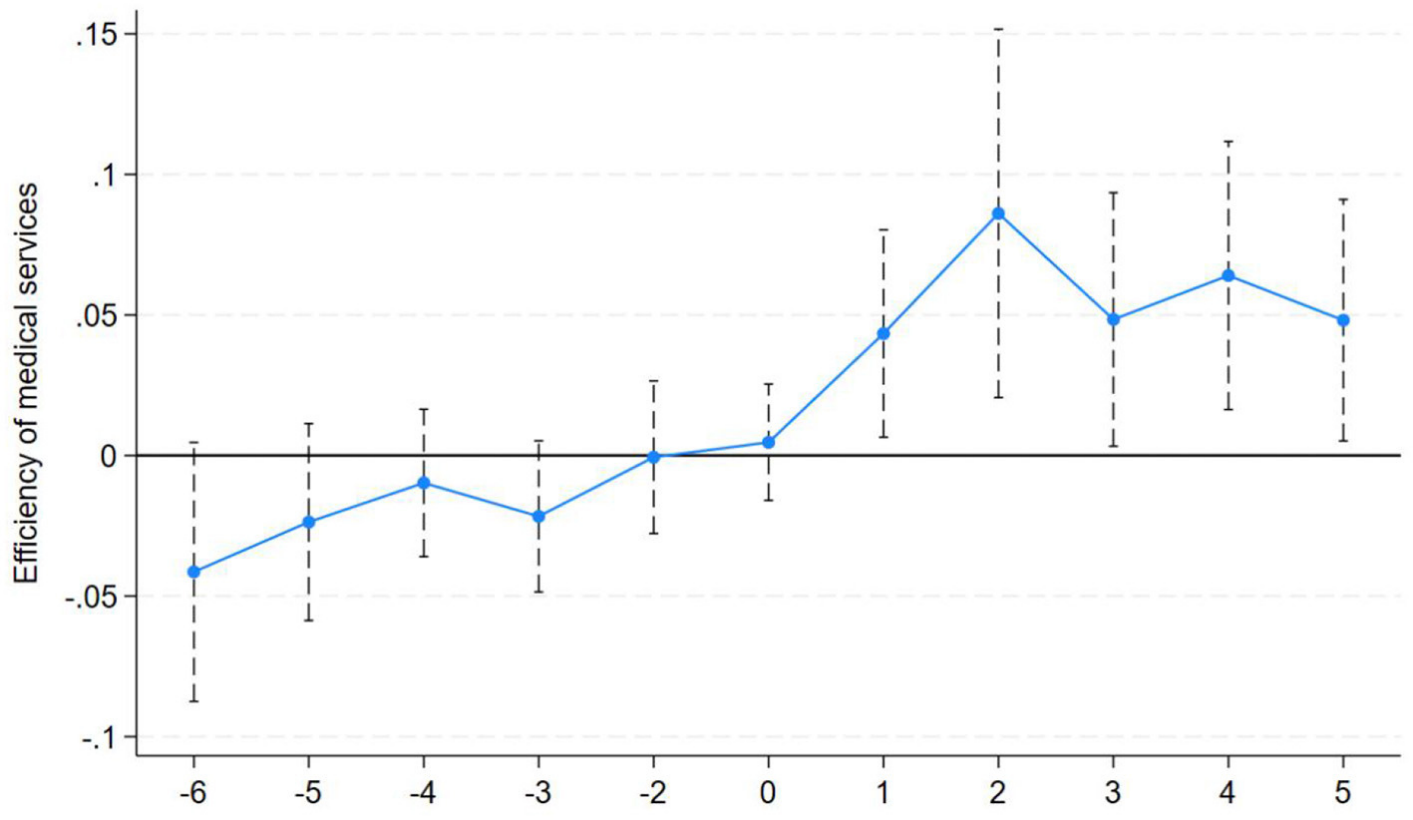
The parallel trends test.

To validate the causal effects of policy implementation, we employ an event study approach to examine the dynamic trends in regional healthcare efficiency before and after the introduction of the LTCI pilot policy. To ensure unbiased regression results, the year before LTCI policy implementation is designated the baseline year. The parallel trends test results are illustrated in Figure 2. As shown, the estimated coefficients for all periods before policy implementation are statistically insignificant and fluctuate around zero, indicating no significant differences in healthcare efficiency between the treatment and control groups. This satisfies the fundamental requirement of the parallel trend assumption. After policy implementation, the coefficients for all periods are significantly positive, demonstrating that the LTCI policy shock has a sustained and significant positive effect on healthcare efficiency in pilot cities, with effects persisting for five periods. The study sample passes the parallel trends test.

### 4.4 Robustness tests

#### 4.4.1 Placebo test

**Figure 3:**
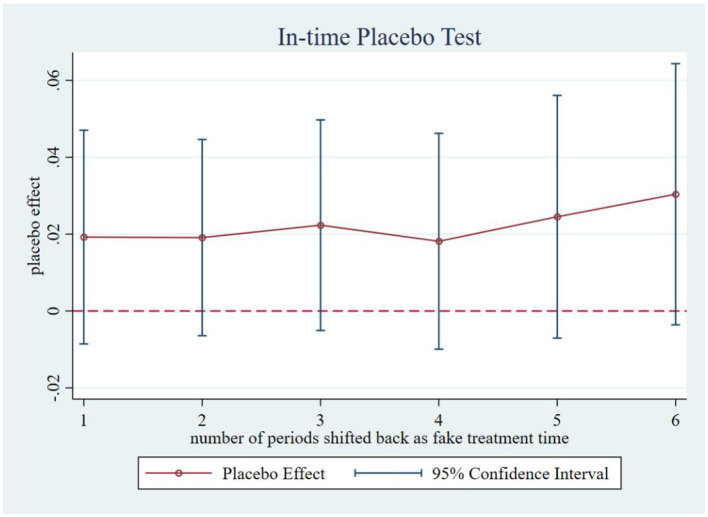
In-time placebo test.

**Figure 4:**
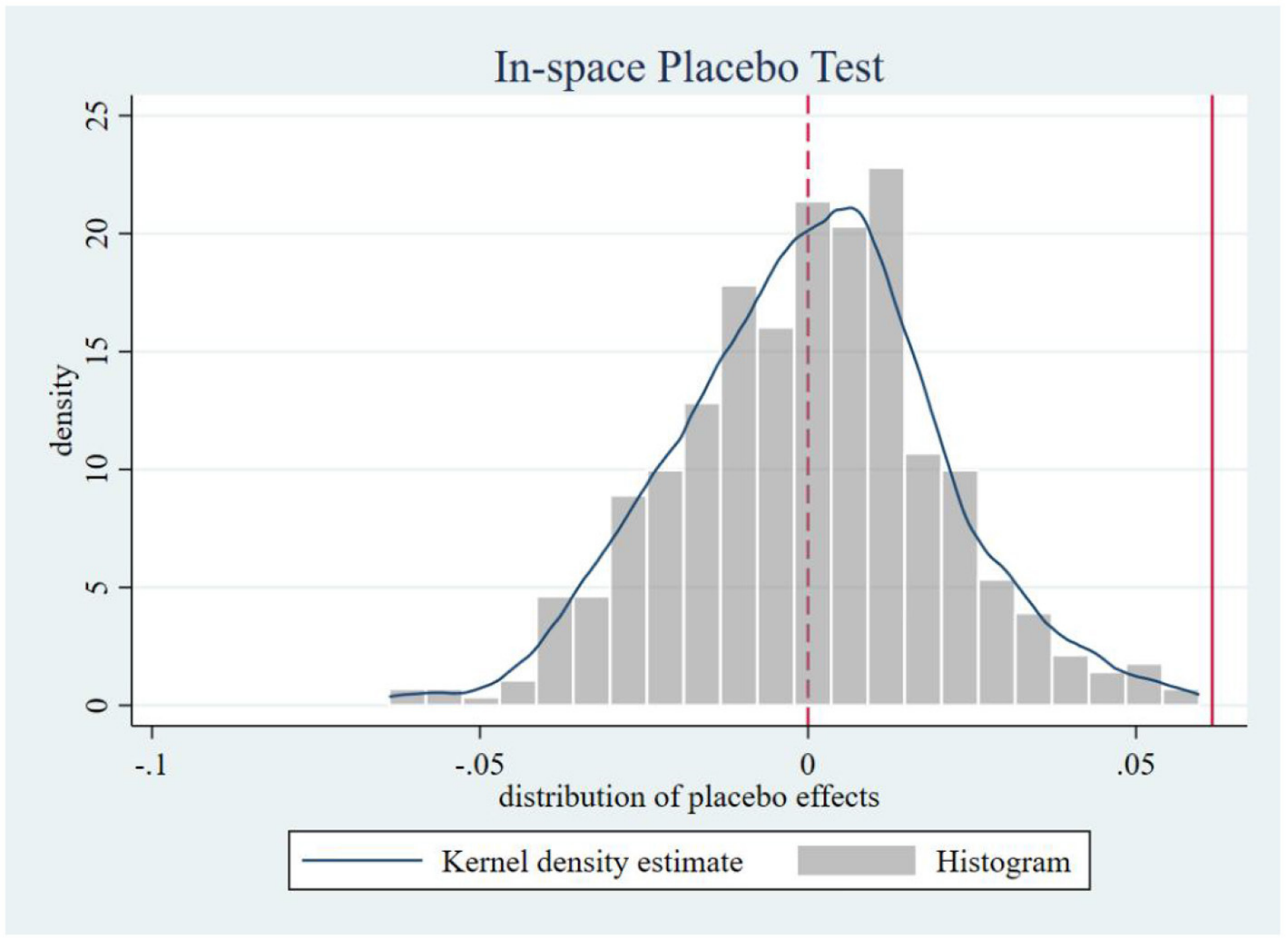
In-space placebo test.

**Figure 5:**
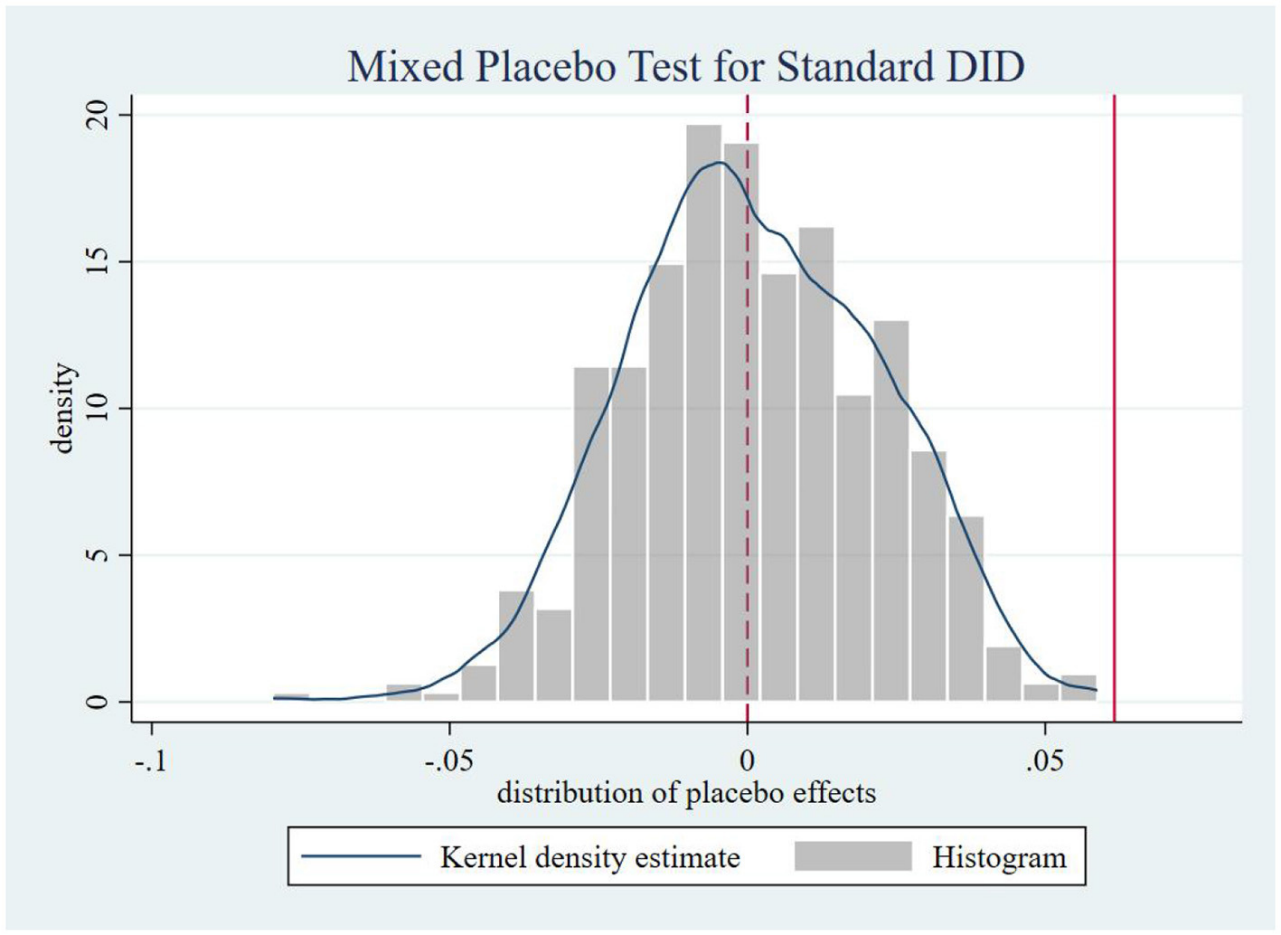
Mixed placebo test.

**Figure 6:**
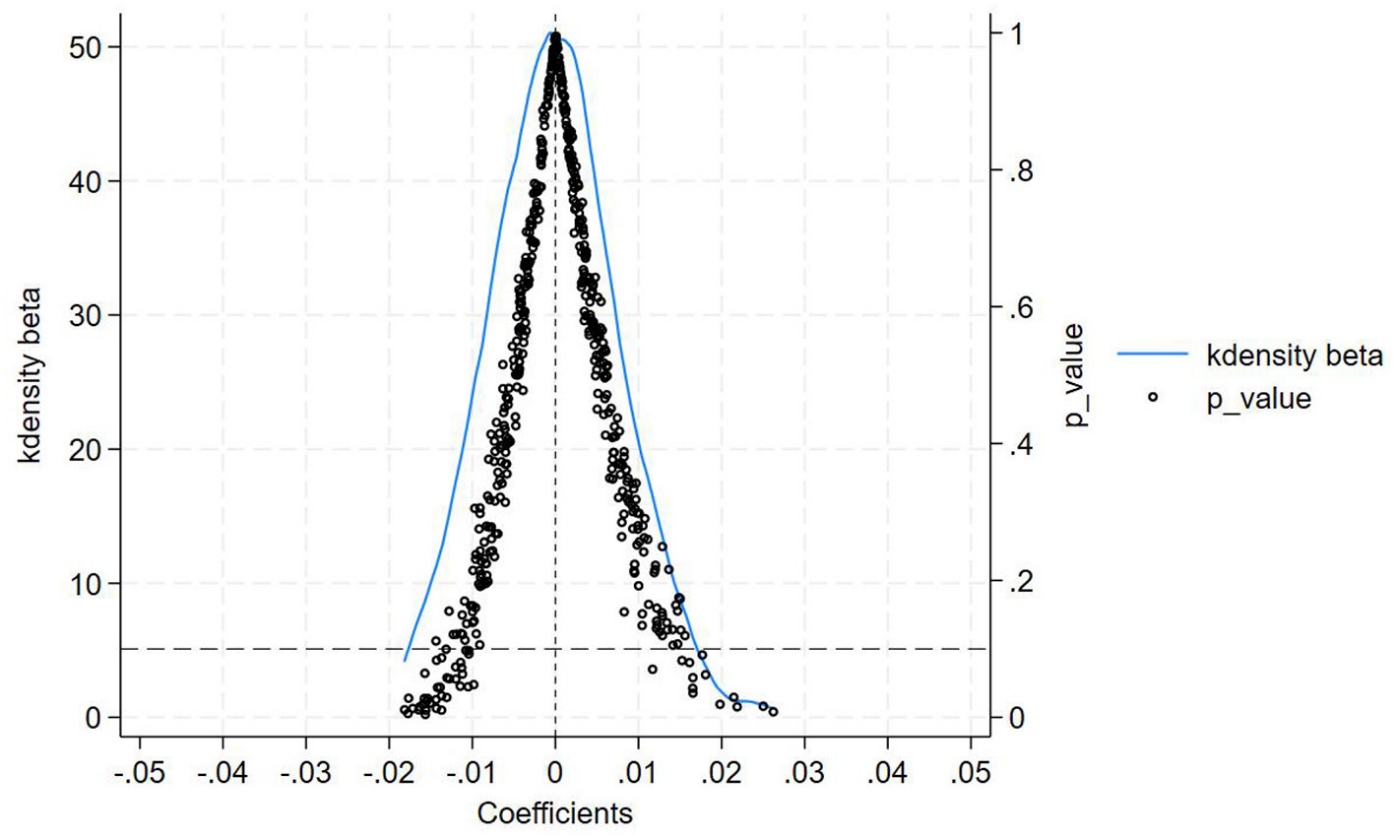
Placebo test.

Following Chen (48), we systematically conducted four placebo tests to mitigate potential spatiotemporal confounding. Firstly, in-time placebo test. We constructed counterfactual policy implementation timelines to generate the effect distribution of the interaction term ltci_post. As shown in Figure 3, when the treatment timing was fictitiously shifted backward by 1 to 6 periods, none of the placebo effect estimates at these artificial treatment points reached statistical significance. This confirms that our core estimates remain unaffected by temporal trends, demonstrating that the observed effects genuinely stem from the treatment intervention rather than time-related confounding factors, thereby reinforcing the reliability of our findings. Secondly, an in-space placebo tests. We maintained the actual policy implementation timeline while conducting 500 randomized treatment group assignments. Figure 4 reveals that the simulated placebo effects follow an approximately symmetric distribution centered around zero, indicating that estimated effects cluster near zero under randomized placebo conditions without systematic bias. Notably, the actual effect size (0.071) lies at the right tail of this distribution, providing evidence that treatment group selection was not influenced by unobserved characteristics. This result effectively rules out estimation biases caused by spatial autocorrelation or other spurious factors, strengthening the validity of our causal inference. Thirdly, a mixed placebo test. We simultaneously randomized both policy timing and treatment group allocation to account for potential spatiotemporal confounding effects. As shown in Figure 5, the effect distribution under this combined fictional scenario again fails to encompass the true effect estimate, further corroborating the robustness of our conclusions. Fourth, a placebo test with randomized treatment–control assignment was implemented to validate robustness. The counterfactual analysis confirms that the efficiency-enhancing impact of LTCI persists, ruling out confounding by unobserved stochastic factors. Figure 6 presents the kernel density distributions of the coefficients from 500 randomly generated treatment groups. As shown, the estimated coefficients from random assignments are concentrated around zero, with most *p-values* exceeding 0.1. The mean of the placebo coefficients is close to zero, significantly lower than the true estimated coefficient of 0.071 from the baseline regression. These findings suggest that the efficiency-enhancing effects of LTCI are unlikely to be attributable to unobserved random factors, strengthening the robustness of the empirical evidence.

#### 4.4.2 Propensity score matching-DID (PSM-DID)

To mitigate potential selection bias in the choice of LTCI pilot cities, we employ a PSM-DID approach, following established methodologies (49). First, we use the PSM method, treating control variables as matching variables, and estimate propensity scores via a logit model with nearest-neighbor matching to assign weights. The balance test results, presented in Table 6, show that the standardized biases for all characteristic variables decrease after matching. The *t*-test results indicate no systematic differences between the treatment and control groups post-matching, suggesting that the matched samples are well balanced and that the matching process is valid and effective.

**Table 6:** The balance test results before and after PSM matching.

**Variable**		**Mean**		**%bias**	***t*** **-test**	
		**Treated**	**Treated**		***t*** **-value**	***p***>**|t|**
Economic development level	Unmatched	11.177	10.676	91.7	10.62	0.000
	Matched	11.177	11.151	4.9	0.45	0.654
Population density	Unmatched	6.143	5.744	47.9	5.42	0.000
	Matched	6.143	6.245	−12.2	−1.25	0.211
Industrial structure	Unmatched	0.917	0.876	62.1	6.55	0.000
	Matched	0.917	0.909	12.5	1.12	0.262
Government fiscal expenditure	Unmatched	0.169	0.203	−36.4	−3.90	0.000
	Matched	0.169	0.165	4.1	0.47	0.639
Education level	Unmatched	0.026	0.019	25.2	3.10	0.002
	Matched	0.026	0.027	−6.0	−0.48	0.632
Openness to trade	Unmatched	0.282	0.199	26.2	2.93	0.003
	Matched	0.282	0.283	−0.5	−0.04	0.969
Urbanization level	Unmatched	0.640	0.548	69.9	7.47	0.000
	Matched	0.640	0.629	8.1	0.76	0.451

We conduct a DID estimation based on the propensity score-matched samples. As shown in Column (1) of Table 7, the coefficient of the explanatory variable “LTCI” remains significantly positive, which is consistent with the baseline regression results.

**Table 7:** Robustness tests.

**Variable**	**(1) (95% CI)**	**(2) (95% CI)**	**(3) (95% CI)**	**(4) (95% CI)**	**(5) (95% CI)**	**(6) (95% CI)**
LTCI	0.190^*^ (−0.002 to 0.381)	0.047^*^ (−0.004 to 0.098)	0.044^**^ (−0.063 to −0.002)	0.071^***^ (0.037 to 0.105)	0.072^***^ (0.035 to 0.108)	0.071^***^ (0.037 to 0.105)
	(0.097)	(0.026)	(0.017)	(0.017)	(0.019)	(0.017)
Hierarchical medical system policy				0.004 (−0.023 to 0.032)		
				(0.014)		
Economic development level	0.066^**^ (0.003 to 0.128)	0.014 (−0.007 to 0.035)	0.044 (−0.012 to 0.104)	0.062^**^ (0.004 to 0.119)	0.059^*^ (−0.009 to 0.128)	0.064^**^ (0.007 to 0.121)
	(0.032)	(0.011)	(0.030)	(0.029)	(0.035)	(0.029)
Density	−0.097 (−0.280 to 0.083)	−0.041 (−0.126 to 0.043)	−0.216^*^ (−0.48 to −0.005)	−0.062 (−0.249 to 0.124)	−0.071 (−0.249 to 0.108)	−0.059 (−0.246 to 0.128)
	(0.093)	(0.043)	(0.123)	(0.095)	(0.091)	(0.095)
Industrial structure	0.357^**^ (0.027 to 0.687)	0.196^**^ (0.046 to 0.345)	−0.688^***^ (−1.17 to −0.21)	0.396^**^ (0.072 to 0.72)	0.333 (−0.075 to 0.741)	0.401^**^ (0.073 to 0.729)
	(0.168)	(0.076)	(0.245)	(0.165)	(0.207)	(0.167)
Government fiscal expenditure	0.254^*^ (−0.033 to 0.541)	0.029 (−0.033 to 0.09)	0.337^***^ (0.131 to 0.583)	0.269^*^ (−0.012 to 0.551)	0.223 (−0.073 to 0.519)	0.272^*^ (−0.014 to 0.558)
	(0.146)	(0.031)	(0.109)	(0.143)	(0.151)	(0.145)
Education level	0.144 (−0.805 to 1.092)	0.114 (−0.352 to 0.58)	1.195^*^ (−0.06 to 2.298)	0.132 (−0.952 to 1.215)	−0.279 (−1.963 to 1.405)	0.123 (−0.974 to 1.219)
	(0.482)	(0.237)	(0.613)	(0.550)	(0.855)	(0.557)
Openness to trade	0.088^**^ (0.021 to 0.156)	−0.016 (−0.053 to 0.022)	−0.003 (−0.082 to 0.058)	0.087^***^ (0.025 to 0.15)	0.076^*^ (−0.002 to 0.153)	0.088^***^ (0.027 to 0.149)
	(0.034)	(0.019)	(0.036)	(0.032)	(0.039)	(0.031)
Urbanization level	0.046 (−0.008 to 0.177)	−0.118^**^ (−0.208 to −0.028)	−0.170^**^ (−0.357 to −0.033)	0.031 (−0.099 to 0.161)	−0.022 (−0.169 to 0.126)	0.033 (−0.095 to 0.16)
	(0.066)	(0.046)	(0.081)	(0.066)	(0.075)	(0.065)
General public budget health expenditure						−0.003 (−0.017 to 0.012)
						(0.007)
Constant	−0.075 (−1.388 to 1.238)	0.175 (−0.335 to 0.685)	2.091^***^ (0.771 to 3.693)	−0.261 (−1.571 to 1.048)	−0.093 (−1.398 to 1.213)	−0.308 (−1.63 to 1.015)
	(0.667)	(0.259)	(0.752)	(0.666)	(0.663)	(0.672)
City	Yes	Yes	Yes	Yes	Yes	Yes
Year	Yes	Yes	Yes	Yes	Yes	Yes
*N*	3,050	3,349	3,349	3,349	2,815	3,349
*R^2^*	0.158	0.139	0.431	0.166	0.153	0.166

^*^p < 0.1, ^**^p < 0.05, ^***^p < 0.01.

#### 4.4.3 Replacing the dependent variable (Super-SBM model under constant returns to scale)

To ensure the robustness of our findings, we reestimate regional healthcare efficiency via the Super-SBM model under constant returns to scale as an alternative to the variable returns to scale assumption in the baseline regression analysis. This alternative approach addresses potential concerns regarding the sensitivity of efficiency measurement to scale-related assumptions. As shown in Column (2) of Table 7, the significance and direction of the coefficients are consistent across both models. This confirms that the impact of LTCI on healthcare efficiency is not driven by scale effects, thereby strengthening the validity of our core conclusions.

#### 4.4.4 Replacing the dependent variable (Replace undesirable output indicators)

To ensure the robustness of regional healthcare efficiency measurements, this study additionally incorporates the dimension of healthcare service quality by substituting the undesirable output indicator with the annual hospitalization rate. After recalculating the efficiency values, the results presented in Column (3) of Table 7 demonstrate that the positive effect of LTCI remains statistically significant (β = 0.044, *P* < 0.05), confirming the robustness of our findings to alternative specifications of undesirable outputs.

#### 4.4.5 Excluding interference from other policies

The hierarchical medical system policy and the LTCI policy may overlap in target populations and service content. Both policies could influence healthcare resource allocation simultaneously, thereby affecting regional healthcare efficiency. Following established methodologies (50), we control for the hierarchical medical system policy variable to isolate its potential confounding effects. As shown in Column (4) of Table 7, after excluding the interference of the hierarchical medical system policy, LTCI continues to significantly improve regional healthcare efficiency (β = 0.071, *P* < 0.01), which is consistent with the baseline regression results.

#### 4.4.6 Shortening the time window

The COVID-19 pandemic in 2020 was a global public health crisis that significantly impacted healthcare resources and service utilization. The utilization rate of routine healthcare services by residents has declined sharply (51, 52), which may have introduced anomalous effects on the relationships among our studied variables. To more accurately measure the routine impact of the LTCI on regional healthcare efficiency, we exclude data from 2020 onward, which were heavily influenced by the pandemic. As shown in Column (5) of Table 7, the LTCI continues to have a significantly positive effect on regional healthcare efficiency (β = 0.072, *P* < 0.01).

#### 4.4.7 Increase control variables

To address potential omitted variable bias, this study incorporates general public budget health expenditure as an additional control variable in robustness tests. This indicator effectively captures the intensity of local government fiscal investment in the healthcare system. As shown in Column (6) of Table 7, the positive effect of the LTCI policy on regional healthcare efficiency remains statistically significant (β = 0.071, *P* < 0.01), with coefficient magnitude highly consistent with baseline estimates. Notably, the hea variable fails to achieve statistical significance, suggesting ambiguous directional effects and weak statistical influence of local government health expenditures on efficiency. These results further confirm that the efficiency-enhancing effect of the LTCI policy remains robust even after accounting for potential confounding from local healthcare fiscal inputs.

### 4.5 Mechanistic analysis

To elucidate the intrinsic mechanisms through which LTCI influences regional healthcare efficiency, this study examined three mediating pathways using a stepwise regression approach combined with the Bootstrap sampling method (with 500 replications). Table 8 presents the mediation effect test results, with detailed analyses as follows:

(1) Mediating effect of hospitalizations. The regression coefficient of LTCI on hospitalization volume was significantly negative (λ = −0.419, *P* < 0.01), which is consistent with the findings of Choi et al. (53). Meanwhile, hospitalization volume exhibited a significantly positive regression coefficient on healthcare efficiency (η = 0.391, *P* < 0.01), suggesting that reduced hospitalization scales improved efficiency by alleviating medical resource congestion. This effect stems from a dual-policy design: first, the differential reimbursement policy stipulates higher coverage rates for home-based and community-based care compared to institutional care, utilizing economic incentives to steer patients toward non-hospital care models. Second, a rigorous dynamic disability assessment mechanism mandates that only patients with disabilities persisting after 6 months of standardized treatment and deemed irreversible are eligible for benefits. This effectively prevents short-term treatment patients from occupying inpatient resources. The reduction in hospitalization demand enhances healthcare efficiency through the following mechanisms: resource reallocation effect. The decline in hospitalization rates liberates bed capacity, allowing preferential allocation to high-value services such as intensive care, thereby reducing the cost of medical resource misallocation (54). The congestion cost reduction effect. The alleviation of hospital overcrowding leads to a measurable decrease in misdiagnosis rates (55), indirectly improving service quality and healthcare efficiency. Table 9 presents the Bootstrap test results for hospitalizations. Using 500 bootstrap resamples and a 95% confidence interval (CI), the analysis revealed a significant indirect effect of 0.002144 [95% percentile CI (0.000053, 0.004924); bias-corrected CI (0.000181, 0.005317)]. Since neither confidence interval included zero, the mediating effect of hospitalization volume was statistically significant. The direct effect remained significant at 0.060165 (*p* < 0.01), confirming that hospitalization volume served as a partial mediator, accounting for 3.42% of the total effect. Thus, Hypothesis H2 was supported.
(2) Mediating effect of average length of hospital stays. Table 8 demonstrated that LTCI significantly reduced the length of hospital stay (λ = −0.326, *P* < 0.01), attributable to decreased patient dependency on hospitalization under LTCI coverage. Furthermore, the shortened hospitalization duration positively influenced healthcare efficiency (η = 0.453, *P* < 0.01). The compression of hospitalization duration accelerates bed turnover rates and reduces the risk of hospital-acquired infections, aligning with the principles of value-based healthcare (56). The policy framework is supported by the following measures: select pilot cities (e.g., Jingmen, Rizhao) mandate designated care institutions to establish dedicated medical care wards, providing 24-h long-term nursing services to eligible beneficiaries, thereby directly substituting hospitalization demand. Additionally, a hospital-to-care-facility referral mechanism has been instituted, facilitating seamless post-discharge transitions through electronic medical record sharing and dedicated liaison officers, effectively reducing inpatient stays. The reduction in hospitalization duration improves healthcare efficiency through two key pathways: first, accelerated bed turnover. Patients with long-term disabilities typically occupy beds significantly longer than those with acute conditions. Shortening hospitalization cycles increases annual bed turnover rates, thereby enhancing service capacity (57). Second, mitigation of nosocomial infection risks. Hospitalization duration exhibits a positive correlation with healthcare-associated infection rates (58). Reduced inpatient stays consequently lower readmission rates due to complications. The bootstrap analysis presented in Table 9 shown that hospital stays functioned as a partial mediator with a substantial contribution rate of 47.6%. These results provide empirical support for Hypothesis H3.
(3) Mediating effect of the number of care institutions. Table 8 indicates that LTCI significantly promoted the development of care institutions (λ = 0.330, *P* < 0.05), demonstrating its incentive effect on the supply side. Furthermore, the expansion of institutional capacity significantly enhanced service efficiency (η = 0.708, *P* < 0.01). The policy's operational efficacy is underpinned by two key components: first, the fund utilization guidelines explicitly mandate earmarked financing for nursing service reimbursements, establishing stable market expectations. Second, pilot regions such as Shanghai, Suzhou, and Tsingtao have integrated commercial insurance institutions into operational roles (e.g., eligibility assessments and claims processing), creating a public-private governance model that accelerates the scaling of care institutions. The expansion of care institutions enhances healthcare efficiency through the following mechanisms: the spatial substitution effect. Increased density of nursing facilities improves service accessibility, elevating the probability of non-hospital care utilization and reducing inefficient hospitalization demand. Economies of scale effect. Institutional proliferation lowers per-bed operational costs, freeing fiscal resources for technological upgrades (59). The bootstrap analysis in Table 9 revealed a statistically significant positive indirect effect through care institutions. The persistent significance of the direct effect indicates that the number of care institutions served as a partial mediator, accounting for 9.23% of the total effect. These findings provide empirical support for Hypothesis H4.

**Table 8:** Mechanistic analysis.

**Variable**	**Hospitalizations**	**Healthcare efficiency**	**Hospital stays**	**Healthcare efficiency**	**Care institutions**	**Healthcare efficiency**
LTCI	−0.419^***^ (−0.621 to −0.217)		−0.326^***^ (−0.344 to −0.308)		0.330^**^ (0.04 to 0.62)	
	(0.103)		(0.009)		(0.147)	
Hospitalizations		0.391^***^ (0.226 to 0.555)				
		(0.084)				
Hospital stays				0.453^***^ (0.411 to 0.496)		
				(0.022)		
Care institutions						0.708^***^ (0.387 to 1.028)
Economic development level	0.048 (−0.044 to 0.139)	0.084^*^ (−0.003 to 0.171)	0.460^***^ (0.43 to 0.491)	0.481^***^ (0.443 to 0.519)	0.160 (−0.086 to 0.405)	0.078 (−0.161 to 0.317)
	(0.047)	(0.044)	(0.015)	(0.019)	(0.125)	(0.122)
Population density	0.082 (−0.372 to 0.536)	0.205 (−0.28 to 0.69)	1.251^***^ (1.153 to 1.35)	1.359^***^ (1.21 to 1.507)	0.679 (−0.644 to 2.003)	0.661 (−0.722 to 2.044)
	(0.231)	(0.246)	(0.050)	(0.076)	(0.673)	(0.703)
Industrial structure	0.779^**^ (0.101 to 1.457)	0.730^*^ (−0.055 to 1.515)	2.374^***^ (2.204 to 2.544)	2.279^***^ (2.064 to 2.495)	2.720^**^ (0.537 to 4.902)	2.376^**^ (0.259 to 4.492)
	(0.344)	(0.399)	(0.087)	(0.109)	(1.109)	(1.075)
Government fiscal expenditure	−0.149 (−0.405 to 0.106)	−0.199 (−0.492 to 0.094)	0.872^***^ (0.724 to 1.021)	0.795^***^ (0.702 to 0.889)	0.332 (−0.201 to 0.865)	0.108 (−0.378 to 0.594)
	(0.130)	(0.149)	(0.076)	(0.048)	(0.271)	(0.247)
Education level	−0.086 (−1.926 to 1.754)	0.279 (−1.559 to 2.118)	1.995^***^ (1.422 to 2.567)	2.269^***^ (1.629 to 2.908)	5.038 (−4.422 to 14.498)	4.685 (−4.932 to 14.301)
	(0.935)	(0.934)	(0.291)	(0.325)	(4.807)	(4.886)
Openness to trade	0.094 (−0.064 to 0.252)	0.010 (−0.079 to 0.279)	0.222^***^ (0.189 to 0.255)	0.214^***^ (0.161 to 0.267)	0.084 (−0.348 to 0.516)	−0.002 (−0.44 to 0.436)
	(0.080)	(0.091)	(0.017)	(0.027)	(0.219)	(0.222)
Urbanization level	−0.037 (−0.361 to 0.286)	−0.242 (−0.547 to 0.062)	2.412^***^ (2.343 to 2.481)	2.244^***^ (2.126 to 2.361)	−1.357^***^ (−2.308 to −0.406)	−1.258^***^ (−2.189 to −0.326)
	(0.164)	(0.155)	(0.035)	(0.060)	(0.483)	(0.473)
Constant	11.22^***^ (8.463 to 13.985)	10.11^***^ (7.343 to 12.884)	−6.916^***^ (−7.607 to −6.226)	−7.769^***^ (−8.658 to −6.88)	1.903 (−6.096 to 9.903)	2.849 (−5.553 to 11.251)
	(1.403)	(1.408)	(0.351)	(0.452)	(4.064)	(4.269)
City	Yes	Yes	Yes	Yes	Yes	Yes
Year	Yes	Yes	Yes	Yes	Yes	Yes
*N*	3,349	3,349	3,349	3,349	3,349	3,349
*R^2^*	0.492	0.449	0.969	0.958	0.054	0.063

^*^p < 0.1, ^**^p < 0.05, ^***^p < 0.01.

**Table 9:** Bootstrap test of three mediating variables.

**Mediating variables**	**Effect**	**Coefficient**	**Bias**	**Std. err**.	**95% conf. interval**
Hospitalizations	Indirect	0.002144	−0.000035	0.001242	0.000053 0.004924 (P)
					0.000181 0.005317 (BC)
	Direct	0.060165	0.000595	0.012480	0.037004 0.085398 (P)
					0.035473 0.085236 (BC)
Hospital stays	Indirect	−0.625359	0.000473	0.012818	−0.649058 −0.598832 (P)
					−0.649591 −0.599043 (BC)
	Direct	0.687668	0.000000	0.000000	0.687668 0.68767 (P)
					0.687668 0.687668 (BC)
Care institutions	Indirect	0.005748	0.000195	0.002472	0.001088 0.011357 (P)
					0.001077 0.011202 (BC)
	Direct	0.056561	0.001331	0.013239	0.032480 0.082837 (P)
					0.030537 0.082718 (BC)

The mediation analysis confirmed three statistically significant pathways through which LTCI improves healthcare service efficiency: reducing hospitalization volume, shortening length of stay, and expanding institutional care supply. Notably, the length of stay reduction demonstrated the strongest mediating effect, highlighting care duration optimization as the pivotal pathway. While significant, the mediating effect through care institution expansion showed relatively weaker magnitude, suggesting its more gradual, long-term nature in efficiency improvement.

### 4.6 Heterogeneity analysis

#### 4.6.1 Heterogeneity analysis by region

Regional heterogeneity is a critical factor influencing policy effectiveness, as different regions present varying levels of healthcare resource availability and healthcare demand (60). Following established methodologies (61), we divide the sample cities into three regional groups—eastern, central, and western—and conduct subgroup regressions. Table 10 shows that LTCI's efficiency-enhancing effects are significantly stronger in eastern (β = 0.063, *P* < 0.01) and central regions (β = 0.113, *P* < 0.01) compared to western (β = 0.162, *P* < 0.01). This regional heterogeneity may stem from systematic disparities in resources, policy implementation, and cultural factors. First, regarding resource endowments, eastern regions exhibit significantly higher nursing facility coverage rates, and tertiary hospital density than western areas, facilitating more effective LTCI resource reallocation. Conversely, western regions face unstable nursing service provision due to primary healthcare workforce attrition. Second, policy implementation quality differs markedly. Eastern local governments outperform their western counterparts in policy innovation indices and fiscal matching rates, ensuring more effective LTCI execution. Some western municipalities demonstrate fund diversion and perfunctory implementation, attenuating policy effectiveness. Third, cultural-behavioral mediators play a role. The prevailing family caregiving tradition in rural western China suppresses formal care demand (62), resulting in lower LTCI service utilization. Religious and ethnic customs further influence service acceptability.

**Table 10:** Heterogeneity analysis by region.

**Variable**	**Eastern (95% CI)**	**Central (95% CI)**	**Western (95% CI)**
LTCI	0.063^***^ (0.021 to 0.105)	0.113^***^ (0.067 to 0.159)	0.162^**^ (0.035 to 0.29)
	(0.021)	(0.023)	(0.064)
Economic development level	0.115^**^ (0.022 to 0.209)	0.036 (−0.033 to 0.105)	0.009 (−0.076 to 0.093)
	(0.047)	(0.035)	(0.043)
Population density	0.024 (−0.288 to 0.336)	0.035 (−0.214 to 0.283)	−0.043 (−0.4 to 0.314)
	(0.157)	(0.125)	(0.180)
Industrial structure	−0.195 (−0.934 to 0.543)	0.407^**^ (0.064 to 0.75)	0.556^*^ (−0.003 to 1.115)
	(0.372)	(0.173)	(0.281)
Government fiscal expenditure	0.027 (−0.085 to 0.138)	0.173 (−0.054 to 0.399)	0.588^***^ (0.188 to 0.987)
	(0.056)	(0.114)	(0.201)
Education level	−2.021 (−5.958 to 1.915)	0.662 (−0.733 to 2.057)	0.658 (−0.505 to 1.822)
	(1.984)	(0.703)	(0.586)
Openness to trade	0.084^*^ (−0.007 to 0.175)	0.147 (−0.202 to 0.497)	0.043 (−0.018 to 0.103)
	(0.046)	(0.176)	(0.031)
Urbanization level	0.081 (−0.157 to 0.319)	0.062 (−0.121 to 0.245)	0.087 (−0.104 to 0.277)
	(0.120)	(0.092)	(0.096)
Constant	−0.728 (−3.118 to 1.661)	−0.693 (−2.375 to 0.99)	−0.005 (−2.41 to 2.4)
	(1.204)	(0.848)	(1.211)
City	Yes	Yes	Yes
Year	Yes	Yes	Yes
*N*	1,190	1,175	984
*R^2^*	0.213	0.221	0.225

^*^p < 0.1, ^**^p < 0.05, ^***^p < 0.01.

#### 4.6.2 Heterogeneity in urban economic development levels

This study adopts the urban classification system from the 2019 China City Commercial Charm Ranking, stratifying prefecture-level cities into six distinct categories based on their commercial development levels: first-tier, new first-tier, second-tier, third-tier, fourth-tier, and fifth-tier cities. Table 11 demonstrates significant heterogeneity in the effectiveness of the LTCI across cities with different economic development levels. The analysis reveals statistically significant effects of LTCI in new first-tier cities (β = 0.088, *P* < 0.01) and third-tier cities (β = 0.098, *P* < 0.05), but non-significant effects in other city tiers. For new first-tier cities, three synergistic factors explain the pronounced policy effects: (1) Structural demand-resource alignment. These cities experience concurrent acceleration in GDP growth and aging rates, enabling LTCI to effectively address structural healthcare gaps. (2) Technological infrastructure. Their expanded coverage of smart eldercare platforms facilitates precise service matching. (3) Policy experimentation advantage. Most serve as pilot cities for healthcare reforms, benefiting from flexible policy innovation privileges. Third-tier cities show significant efficiency gains primarily through marginal improvement mechanisms, where LTCI-introduced resources and concepts generate substantial impacts due to their lower baseline development levels (63). The lack of significant effects in first-tier cities likely stems from market saturation and excessive competition diminishing marginal policy returns, while fifth-tier cities' constrained administrative capacity and fiscal fragility impair effective policy implementation. These findings highlight the importance of considering urban developmental stages when evaluating health policy effectiveness.

**Table 11:** Heterogeneity analysis by economic development.

**Variable**	**First-tier (95% CI)**	**New first-tie (95% CI)**	**Second-tier (95% CI)**	**Third-tier (95% CI)**	**Fourth-tier (95% CI)**	**Fifth-tier (95% CI)**
LTCI	0.001 (−0.109 to 0.112)	0.088^***^ (0.034 to 0.142)	0.028 (−0.046 to 0.102)	0.098^**^ (0.021 to 0.174)	0.048 (−0.019 to 0.116)	0.076 (−0.099 to 0.251)
	(0.035)	(0.027)	(0.036)	(0.039)	(0.034)	(0.082)
Economic development level	0.135^*^ (0 to 0.27)	0.057 (−0.031 to 0.144)	0.047 (−0.062 to 0.155)	0.167^**^ (0.031 to 0.302)	0.053 (−0.074 to 0.181)	−0.123^**^ (−0.22 to −0.027)
	(0.043)	(0.044)	(0.053)	(0.068)	(0.064)	(0.045)
Population density	0.736^*^ (−0.103 to 1.576)	−0.242 (−0.615 to 0.132)	−0.454^**^ (−0.805 to −0.103)	0.077 (−0.285 to 0.438)	−0.219 (−0.585 to 0.148)	−0.424 (−1.213 to 0.365)
	(0.264)	(0.187)	(0.172)	(0.182)	(0.184)	(0.368)
Industrial structure	−11.53 (−74.203 to 51.146)	0.241 (−0.572 to 1.053)	2.063^*^ (−0.347 to 4.472)	−0.110 (−0.544 to 0.324)	0.918^***^ (0.278 to 1.558)	7.649 (−3.932 to 19.229)
	(19.69)	(0.407)	(1.178)	(0.219)	(0.322)	(5.399)
Government fiscal expenditure	1.642^*^ (−0.397 to 3.682)	0.025 (−0.059 to 0.108)	0.253 (−0.555 to 1.062)	0.599^***^ (0.19 to 1.009)	0.440^**^ (0.036 to 0.844)	0.550 (−1.431 to 2.531)
	(0.641)	(0.042)	(0.395)	(0.206)	(0.203)	(0.924)
Education level	25.63^***^ (11.713 to 39.539)	−0.251 (−2.208 to 1.706)	−0.313 (−1.892 to 1.265)	0.790 (−0.59 to 2.17)	1.194 (−1.238 to 3.626)	−5.351 (−18.576 to 7.875)
	(4.372)	(0.981)	(0.772)	(0.695)	(1.222)	(6.166)
Openness to trade	0.256^**^ (0.01 to 0.502)	0.061 (−0.074 to 0.195)	0.226^***^ (0.152 to 0.3)	0.048 (−0.021 to 0.117)	0.395^***^ (0.16 to 0.63)	0.031 (−0.181 to 0.243)
	(0.077)	(0.067)	(0.036)	(0.035)	(0.118)	(0.099)
Urbanization level	0.623^*^ (−0.101 to 1.346)	−0.249^**^ (−0.468 to −0.03)	0.729^***^ (0.21 to 1.247)	0.034 (−0.156 to 0.223)	0.104 (−0.077 to 0.285)	−0.216 (−0.749 to 0.317)
	(0.227)	(0.110)	(0.253)	(0.095)	(0.091)	(0.249)
Constant	2.833 (−65.985 to 71.654)	1.173 (−1.273 to 3.62)	0.289 (−2.183 to 2.761)	−1.668 (−4.257 to 0.922)	0.172 (−2.204 to 2.548)	−2.449 (−15.557 to 10.659)
	(21.62)	(1.226)	(1.209)	(1.304)	(1.194)	(6.111)
City	Yes	Yes	Yes	Yes	Yes	Yes
Year	Yes	Yes	Yes	Yes	Yes	Yes
*N*	47	828	359	1,023	912	180
*R^2^*	0.821	0.226	0.461	0.208	0.210	0.235

^*^p < 0.1, ^**^p < 0.05, ^***^p < 0.01.

## 5 Discussion

This study utilizes panel data from 291 prefecture-level cities in China (2010–2021) and employs the DID method, the Super-SBM model, and mediation effect models to systematically examine the impact of the LTCI on healthcare efficiency. The baseline regression results indicate that LTCI significantly improves regional healthcare efficiency (β = 0.071, *P* < 0.01), which is consistent with the findings of Li et al. (64). LTCI promotes the utilization of healthcare services and enhances healthcare efficiency (13, 19).

In the mechanism analysis, we integrate Grossman's health demand theory and Arrow's healthcare supply structure remodeling theory to construct a comprehensive demand substitution-supply inducement analytical framework. The Grossman model's price and income effects manifest distinctly in LTCI policy implementation. Differential reimbursement policies significantly reduce the relative price of nursing services, as evidenced by higher coverage ratios for home/community-based care vs. institutional care in most cities (e.g., Shanghai, Chengde) (65). This price effect directly enhances the marginal substitution rate between nursing services and hospitalization (Table 8). Simultaneously, LTCI's income effect alleviates household budget constraints, effectively reducing excessive hospitalizations caused by catastrophic medical expenditures. From the supply-side perspective, Arrow's theoretical framework highlights the formative influence on provider behavior. China's LTCI system achieves hospitalization duration reduction through institutional designs, including per-diem payment caps and referral protocols. These findings suggest that in transitioning economies with underdeveloped healthcare markets, exogenous institutional constraints can effectively substitute for market mechanisms as primary regulatory tools. Furthermore, the network externality effects from nursing facility expansion reveal mechanisms of supplier-induced demand elasticity, thereby extending Arrow's theoretical framework by incorporating dynamic supply-side evolutionary dimensions. These findings align with the studies of Vrabkov and Lee (67) and Hejazi (66), revealing a unique pathway for efficiency improvement in developing countries under resource constraints. Diverging from the market-oriented approaches prevalent in developed countries (68), China's LTCI system has achieved rapid efficiency improvements under resource constraints through government-led institutional arrangements, including dedicated fund guarantees and public-private partnership in program administration. This institutional contrast suggests that the conventional Grossman-Arrow framework requires incorporation of institutional dimensions when applied to developing contexts, particularly to account for path dependency in policy instrument selection.

Heterogeneity analysis provides critical insights for the design of policies. The significant effects of the LTCI in the eastern (β = 0.063) and central regions (β = 0.113) validate the resource endowment and policy response theory (69), suggesting that well-developed healthcare infrastructure amplifies policy dividends. The attenuated policy effect observed in western regions may originate from suboptimal policy implementation, inadequate fiscal matching rates, and constrained grassroots administrative capacity (70). These findings underscore the necessity to develop LTCI implementation frameworks tailored to regional developmental stages. Specifically, pilot programs establishing regional care alliances (following the Australian model) (71) could be implemented in western China to overcome service fragmentation through cross-jurisdictional resource integration. Moreover, the dual-peak effects observed in new first-tier (β = 0.088) and third-tier cities (β = 0.098) reveal a policy opportunity window for medium-level cities (72). New first-tier cities (e.g., Chengdu, Wuhan) demonstrate superior policy outcomes attributable to structural alignment advantages. The synchronous acceleration of population aging and GDP growth, coupled with comprehensive smart eldercare platform coverage, enables precise healthcare resource allocation. In contrast, third-tier cities exhibit higher marginal returns due to their baseline resource scarcity. These findings have important implications for low- and middle-income countries: under fiscal constraints, policies should prioritize medium-level development corridor cities to maximize the input–output ratio.

Although this study provides robust evidence that LTCI significantly improves regional healthcare efficiency, it also has several limitations. First, constrained by data availability, our analysis period concludes in 2021. Consequently, the second batch of LTCI pilot cities (e.g., Southwestern Guizhou, Urumqi), initiated in 2020, are excluded from this study, as their implementation details were predominantly operationalized post-2021, resulting in insufficient policy exposure duration. Given the relatively short evaluation period (2016–2021), this study may only reflect the initial-stage effects of the LTCI policy. The long-term impacts, such as the progressive optimization of healthcare resource allocation and the maturation of the care service market, have yet to fully materialize. Consequently, the findings may underestimate the comprehensive efficacy of LTCI. Future research should extend the observation window to properly evaluate these late-implemented programs. Second, while our dual-pathway model elucidates LTCI's direct mechanisms affecting both technical and allocative efficiency of medical resources, it does not capture the healthcare system's full complexity. The exclusion of informal care (e.g., family care) and its potential substitution effects may lead to an overestimation of LTCI's direct impact on hospitalization reduction. If family care and LTCI services exhibit substitution relationships, the actual net policy effect could be lower than current estimates. Future research should incorporate micro-level survey data, such as time costs of family care and service utilization preferences, to quantify this substitution effect more precisely.

## 6 Conclusion and recommendations

In developing countries facing the dual challenges of aging populations and increasing healthcare burdens, LTCI represents a critical solution. This study utilizes panel data from 291 prefecture-level cities in China (2010–2021) and employs the DID method, the Super-SBM model, and mediation effect models to examine the impact of the LTCI on regional healthcare efficiency. The findings reveal that (1) LTCI significantly improves regional healthcare efficiency (β = 0.071, *P* < 0.01). (2) A mechanistic analysis demonstrated that LTCI enhances healthcare efficiency by reducing the number of hospitalizations, shortening the average length of hospital stay, and increasing the supply of care institutions. (3) Heterogeneity analysis indicates that the effects of the LTCI are more pronounced in the eastern and central regions and in new first-tier and third-tier cities. China's LTCI experience, characterized by its dual demand-supply side synergistic reforms and regionally adaptive implementation mechanisms, offers valuable policy design paradigms for developing countries with distinct institutional contexts, such as India (with its strong family care traditions) and Brazil (facing fragmented community care systems). However, successful policy transfer requires careful contextual adaptation to local institutional environments.

This study proposes the following policy recommendations: first, regarding regionally differentiated strategies, we propose three targeted interventions for western China: (1) Establishing cross-jurisdictional care resource-sharing platforms to enable dynamic allocation of institutional beds and professional staff through provincial-level coordination mechanisms. (2) Issuing central government special nursing bonds to specifically fund community care station development in underserved western regions. (3) implementing a hybrid policy combining family caregiver cash allowances with telemedicine support in rural western areas, featuring dedicated subsidies for households providing continuous home care for ≥6 months, integrated with 5 G-enabled teleconsultation systems linking county hospitals to home-based care settings.

Second, to address the bimodal distribution of policy effects across city tiers, we propose differentiated implementation pathways: (1) In new first-tier cities (e.g., Wuhan, Chengdu), smart nursing innovation pilot zones should be established, focusing on developing bundled payment mechanisms for post-acute care that integrate DRG payments with LTCI reimbursement, thereby creating seamless hospital-rehabilitation-nursing care transitions. (2) For third-tier cities (e.g., Urumqi, Weifang), 15-min community nursing accessibility circles should be implemented through earmarked fiscal transfers, ensuring two standardized nursing stations per sub-district. (3) In mature first-tier cities (Beijing, Shanghai), we recommend establishing competitive long-term care service trading platforms coupled with star-rated certification systems for care providers, leveraging market mechanisms to optimize service quality.

Third, for supply-side reforms, we propose three key measures: (1) Mandating all pilot cities to implement facility star-rating systems directly linked to service pricing and insurance reimbursement schedules. (2) Expanding public-private partnerships by engaging commercial insurers in program administration. (3) Optimizing demand-side incentives through tiered cost-sharing mechanisms featuring income-dependent out-of-pocket ceilings for low-income groups and progressive co-payment schedules for middle/high-income households, while integrating informal home care into benefit packages via LTCI-community center hybrid models.

## Data Availability

Publicly available datasets were analyzed in this study. This data can be found here: EPS: https://epschinastats.com/ and China Yearbook of Statistics: https://data.stats.gov.cn/english/publish.htm?sort=1.
